# Super-resolution microscopy reveals the nanoscale cluster architecture of the DEK protein cancer biomarker

**DOI:** 10.1016/j.isci.2023.108277

**Published:** 2023-10-19

**Authors:** Agnieszka Pierzynska-Mach, Alberto Diaspro, Francesca Cella Zanacchi

**Affiliations:** 1 Nanoscopy and NIC@IIT, Istituto Italiano di Tecnologia, 16152 Genoa, Italy; 2 Department of Physics (DIFILAB), Department of Physics, University of Genoa, 16146 Genoa, Italy; 3 Physics Department E. Fermi, University of Pisa, 56127 Pisa, Italy; 4 Centro per l’Integrazione della Strumentazione dell’Università di Pisa (CISUP), University of Pisa, 56127 Pisa, Italy

**Keywords:** Optical imaging, Cell biology, Cancer

## Abstract

DEK protein, a key chromatin regulator, is strongly overexpressed in various forms of cancer. While conventional microscopy revealed DEK as uniformly distributed within the cell nucleus, advanced super-resolution techniques uncovered cluster-like structures. However, a comprehensive understanding of DEK’s cellular distribution and its implications in cancer and cell growth remained elusive. To bridge this gap, we employed single-molecule localization microscopy (SMLM) to dissect DEK’s nanoscale organization in both normal-like and aggressive breast cancer cell lines. Our investigation included characteristics such as localizations per cluster, cluster areas, and intra-cluster localization densities (ICLDs). We elucidated how cluster features align with different breast cell types and how chromatin decompaction influences DEK clusters in these contexts. Our results indicate that DEK’s intra-cluster localization density and nano-organization remain preserved and not significantly influenced by protein overexpression or chromatin compaction changes. This study advances the understanding of DEK’s role in cancer and underscores its stable nanoscale behavior.

## Highlights

• DEK’s nanoscale organization studied via single-molecule localization microscopy
• DEK protein clusters differ in normal-like and cancerous breast cell lines
• Chromatin decompaction influences DEK cluster features
• Intra-cluster localization density (ICLS) of DEK clusters remains consistent

## Graphical abstract

**Figure:**
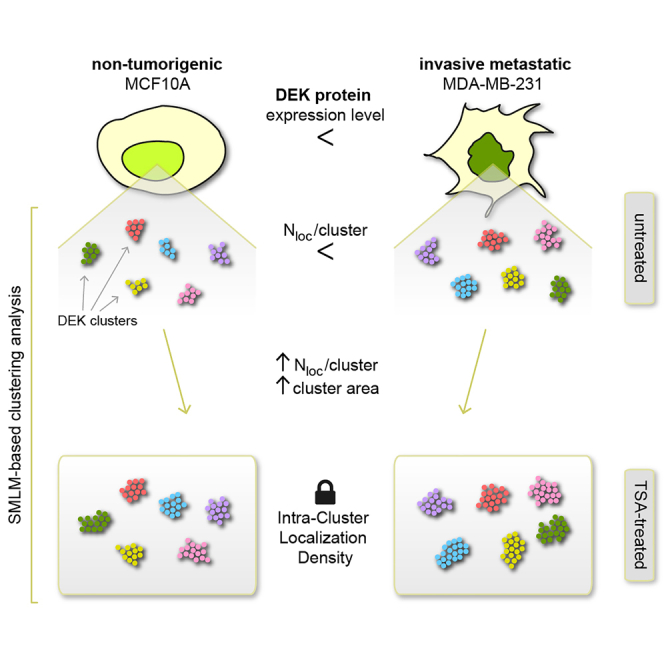

## Introduction

Cancer is one of the leading causes of mortality and can originate from the alteration of genetic material or by heritable features of epigenetic characteristics.^1^ Two critical features in cancer progression are an abnormal chromatin local organization and the perturbation of the nanoscale landscape of the nuclear factors.^2^^,^^3^

One of the non-histone chromatin architectural factors is DEK, which has a pleiotropic mode of action by influencing several regulatory cellular pathways. DEK is involved in chromatin structure and chromatin-related processes regulation, such as transcriptional activation and repression,^4^^,^^5^^,^^6^ DNA replication,^7^^,^^8^^,^^9^^,^^10^ and DNA damage response and repair.^11^^,^^12^^,^^13^^,^^14^^,^^15^ Throughout the cell cycle, DEK exhibits dynamic behavior within the nucleus, as revealed by various studies.^4^^,^^16^^,^^17^^,^^18^^,^^19^ During interphase (comprising G1, S, and G2 phases), DEK is evenly dispersed within chromatin, displaying no strong association with condensed chromosomes.^16^ However, as the cell progresses into mitosis, DEK’s role takes a different turn. During metaphase, it prominently associates with the peripheries and regions of sparse chromatin in condensed chromosomes,^18^ which may suggest that DEK might function as an architectural protein supporting specific chromosomal domains. Late in mitosis, during telophase, DEK re-associates with DNA.^18^ Aberrant DEK overexpression has been linked to its anomalous association with mitotically defective chromosomes, micronuclei formation, and potentially oncogenic mutations, underscoring its role in cancer initiation.^19^ These findings highlight the multifaceted dynamics of DEK protein during different phases of the cell cycle, offering insights into its potential contributions to chromatin organization and cancer development. DEK binds to many highly and commonly expressed genes^5^ and is involved in gene regulation in breast cancer cells.^20^ The protein DEK can be observed mainly inside the cell nucleus of eukaryotic cells,^4^^,^^21^^,^^22^ and several studies demonstrated that it associates with open chromatin, rich in euchromatin histone marks.^5^^,^^18^^,^^22^^,^^23^^,^^24^ However, DEK has also been implicated in the maintenance of transcriptionally inactive heterochromatin.^25^^,^^26^

Interestingly, DEK is highly overexpressed in numerous forms of cancer,^27^^,^^28^^,^^29^^,^^30^^,^^31^^,^^32^^,^^33^ and, in general, its expression is considered an indicator of the cell proliferation level.^34^ In the context of breast cancer, for example, western Blot and immunohistochemistry assays showed that DEK is highly expressed in human breast cell lines (e.g., different MDAMB cell lines), less expressed in a non-tumorigenic immortalized cell line (MCF10A), and expressed the least in normal tissues.^32^^,^^35^ These assays provide critical information on the overall protein expression but clearly cannot report the effects of such overexpression on the precise spatial distribution of the protein in the cell. When observed with diffraction-limited fluorescence microscopy, DEK is commonly described as uniformly distributed within the cell nucleus.^22^^,^^24^^,^^36^ However, our recent study employing super-resolution single-molecule localization microscopy (SMLM) methods based on stochastic optical reconstruction microscopy (STORM)^37^ suggests the formation of cluster-like DEK structures.^10^ In this context, the resolution reachable by STORM^37^ provides a unique opportunity to quantitatively investigate the DEK cluster organization.

In this study, we perform a thorough characterization of such DEK clusters for normal-like (MCF10A) and metastatic (MDAMB231) breast cancer cell lines, known to exhibit a different level of overall DEK expression, through a combination of SMLM and clustering analysis approaches, aiming to uncover the topological landscape of this cancer biomarker.

## Results

### DEK distribution into nanoclusters differs between normal-like and metastatic breast cancer cells

**Figure 1:**
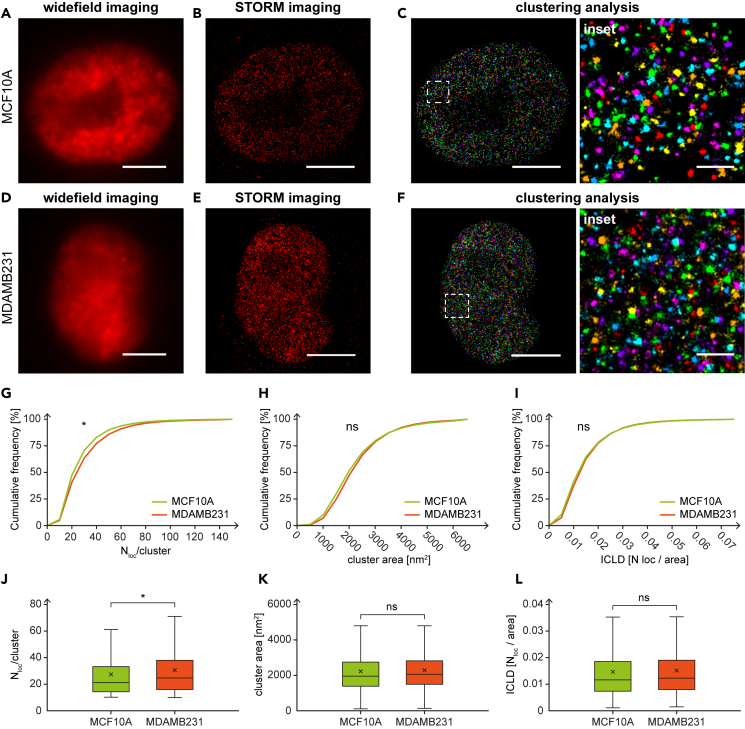
Super-resolution imaging of DEK protein clusters in breast cancer model cell lines (A) Widefield, (B) STORM, and (C) clustering analysis image of DEK protein in MCF10A nucleus immunolabeled with AF647 (each color represents different cluster ID). (D) Widefield, (E) STORM, and (F) clustering image of DEK protein in MDAMB231 nucleus immunolabeled with AF647 (each color represents different cluster ID). Cumulative distributions of (G) the number of localizations/cluster, (H) cluster area, and (I) intra-cluster localization density (ICLD) show the DEK cluster features differences between MCF10A and MDAMB231 cells. Panels (J), (K) and (L) show the boxplot representation of the data distributions of the number of localizations per cluster, cluster areas, and ICLDs, respectively, based on SMLM imaging (description of boxplots in STAR Methods). The average number of localizations per cluster in MCF10A cells: 27.2 ± 18.7 compared to MDAMB231 cells: 30.7 ± 21.9. The average cluster areas MCF10A cells: 2224.9 ± 1252.8 [nm^2^] compared with MDAMB231 cells: 2282.8 ± 1131.1 [nm^2^]. The average ICLDs in MCF10A cells: 0.0147 ± 0.011 [N_loc_/cluster] and MDAMB231 cells: 0.0151 ± 0.0106 [N_loc_/cluster]. The total number of analyzed clusters is: N_MCF10A_ = 26905 and N_MDAMB231_ = 22007. Not significant when p > 0.05; ∗ when p ≤ 0.05. Scale bar: 5 μm. Scale bar insets: 500 nm. See also Figures S2 and S4.

To characterize DEK protein cellular distribution, we used single-molecule-localization-based super-resolution microscopy. This approach allowed us to quantitatively compare the DEK pattern in MCF10A and MDAMB231 cells, representing the normal-like breast basal epithelial cells and triple negative, metastatic basal breast cancer cells, respectively. Previous studies indicate that DEK protein locates mostly within the cell nucleus,^4^^,^^10^^,^^22^ and, more precisely, it can be associated with euchromatin (i.e., the active compartments of chromatin).^18^ Considering the overexpression of DEK in breast cancer samples, we speculated that its nanoscale organization would differ if compared with normal-like cells. To verify this, we studied its cluster organization (Figure 1). MCF10A and MDAMB231 cells were fixed and immuno-stained against DEK following the protocol suitable for fluorescence super-resolution microscopy (STAR Methods). We used stochastic optical reconstruction microscopy (STORM) and clustering analysis to identify DEK protein clusters and characterize the number of localizations per cluster, as well as areas of individual clusters. Widefield images (Figures 1A and 1D) did not reveal major visual differences in the DEK pattern between the cell lines; however, the SMLM imaging (Figures 1B and 1E, Figure S1) and the clustering analysis gave us precise insight into the features of individual DEK clusters. In order to perform the analysis, clusters of DEK were segmented using a previously developed distance-based algorithm^38^ (Figures 1C and 1F). Based on this operation, we obtained the distributions of the number of localizations per cluster (Figure 1G) and of the cluster area (Figure 1H) for each cell type. The average number of localizations per DEK cluster in MDAMB231 cells (30.7 ± 21.9) resulted in about 13% higher than in MCF10A (27.2 ± 18.7) (Figure S2A), and the difference of the distributions was statistically significant (p ≤ 0.05). Furthermore, we observed a higher mean area of DEK clusters in MDAMB231 cells compared with MCF10A cells (about 2.6% higher) (Figure S2B), although the difference was not statistically significant (p = 0.0796). Next, we computed the distributions of the DEK intra-cluster localization densities (ICLD), defined as the number of localizations divided by the cluster area, in both cell lines and observed that they exhibited no statistically significant differences (Figure 1I). This result brought us to hypothesize that the DEK protein organizes in clusters of different number of protein copies across different cancer cell states. Still, the ICLD is conserved, regardless of the DEK expression level.

To investigate if this behavior is a more general trait, we decided to compare the DEK distribution between two different chromatin compaction states. Indeed, it is known that chromatin compaction alters the nanoscale distribution of chromatin components and chromatin-associated proteins.^38^

### The influence of TSA on chromatin decondensation and redistribution of DEK protein in normal-like and metastatic breast cancer cells

In order to control the chromatin compaction, we used Trichostatin A (TSA), a well-known histone deacetylase inhibitor that induces the chromatin opening^39^ and leads to chromatin decompaction by global histone hyperacetylation. The global TSA-induced chromatin relaxation has been shown using traditional confocal microscopy^39^^,^^40^ as well as utilizing the fluorescence lifetime.^41^^,^^42^ Recently, the change in chromatin organization by opening the DNA due to decreased nucleosomal occupancy has been also revealed with SMLM.^38^^,^^43^

**Figure 2:**
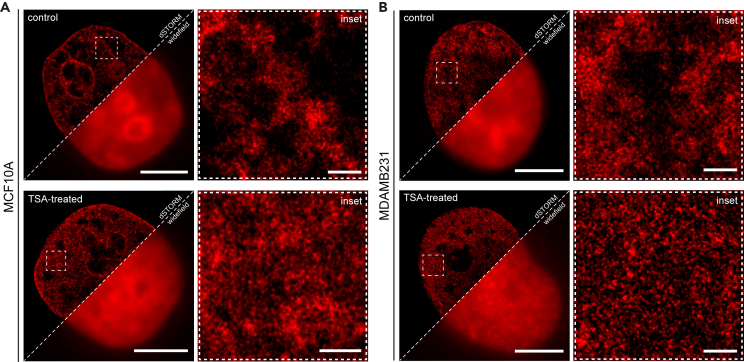
SMLM imaging reveals the influence of TSA on the DNA compaction Widefield and super-resolution images of DNA in the nucleus labeled with EdU (incubation 24h) in MCF10A (A) and MDAMB231 cells (B); scale bar: 5 μm. The insets reflect the euchromatin region within the squares in a full-size image. Scale: 400 nm. See also Figures S1 and S3.

To confirm the TSA influence on chromatin compaction in the selected cell lines, we observed the DNA density using both widefield and SMLM imaging in control and TSA-treated cells (Figure 2). The control samples of both MCF10A (Figure 2A) and MDAMB231 (Figure 2B) exhibit typical DNA distribution within the cell nucleus with higher fluorescence intensity coming from dense, heterochromatin regions (nuclear periphery and surrounding of the nucleolus). Lower-density euchromatin regions exhibit local variations in DNA density (Figures 2A and 2B upper row, insets). The TSA treatment of MCF10A and MDAMB231 cells resulted in the global hyperacetylation of histone tails, which manifested with the genome-wide decompaction of DNA when observed with SMLM (Figures 2A and 2B lower row, insets; Figure S2). We complemented these results with traditional confocal microscopy imaging, which allowed to observe DNA density regions of higher and lower fluorescence intensity (stained with ToPro3) in control cells (Figures S3A and S3C) and hyperacetylated nuclear chromatin in TSA-treated cells (Figures S3B and S3D).

These results are in accordance with previously done studies with the use of SMLM, which have shown the DNA density change upon the influence of TSA in human BJ fibroblasts.^43^ Once confirmed that TSA effectively inflicts chromatin decompaction, we moved to investigate the potential nanoscale redistribution of DEK clusters following the TSA treatment.

**Figure 3:**
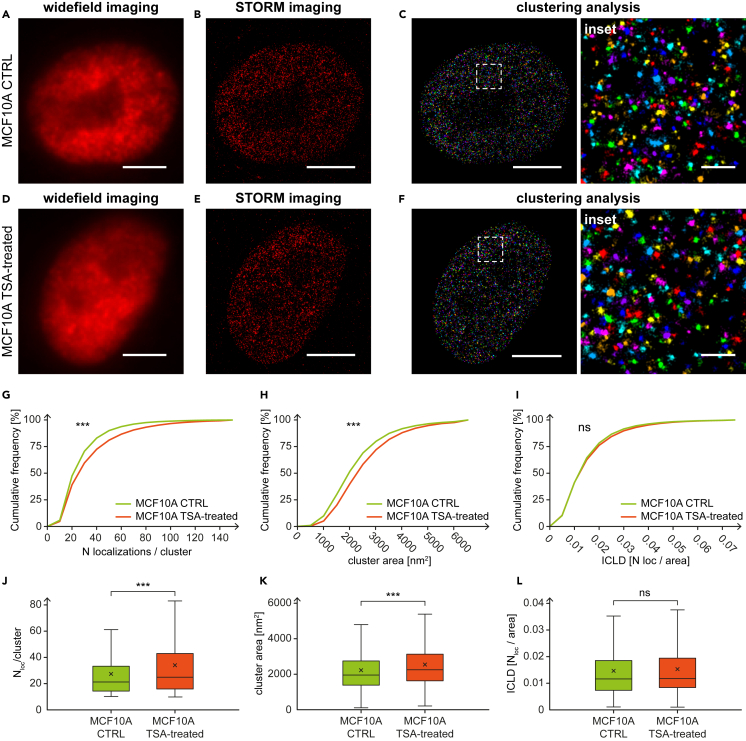
Super-resolution imaging of DEK protein clusters in control and TSA-treated MCF10A cells (A) Widefield, (B) STORM, and (C) clustering image of DEK protein in control nucleus immunolabeled with AF647 (each color represents different cluster ID). (D) Widefield, (E) STORM, and (F) clustering image of DEK protein in TSA-treated nucleus immunolabeled with AF647 (each color represents different cluster ID). Cumulative distributions of (G) the number of localizations/cluster, (H) cluster area, and (I) ICLD show the DEK cluster features differences between control and TSA-treated cells. Panels (J), (K), and (L) show the boxplot representation of the data distributions of the number of localizations per cluster, cluster areas, and ICLD, respectively, based on SMLM imaging. The average number of localizations per cluster in control cells: 27.2 ± 18.7 compared with TSA-treated cells: 34.0 ± 26.3. The average cluster areas control cells: 2224.9 ± 1252.8 [nm^2^] compared with TSA-treated cells: 2543.1 ± 1369.0 [nm^2^]. The average cluster densities in control cells: 0.0147 ± 0.011 [N_loc_/cluster] and TSA-treated cells: 0.0153 ± 0.0118 [N_loc_/cluster]. The total number of analyzed clusters: N_control_ = 26905 and N_TSA-treated_ = 21913. Not significant when p > 0.05; ∗∗∗ when p ≤ 0.001. Scale bar: 5 μm. Scale bar insets: 500 nm. See also Figures S2 and S4.

**Figure 4:**
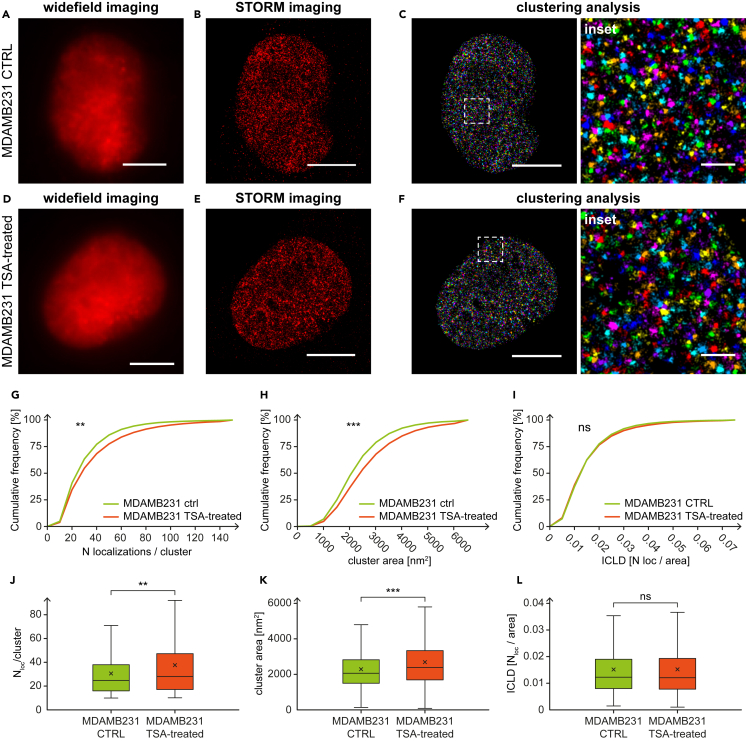
Super-resolution imaging of DEK protein clusters in control and TSA-treated MDAMB231 cells (A) Widefield, (B) STORM, and (C) clustering image of DEK protein in control nucleus immunolabeled with AF647 (each color represents different cluster ID). (D) Widefield, (E) STORM, and (F) clustering image of DEK protein in TSA-treated nucleus immunolabeled with AF647 (each color represents different cluster ID). Cumulative distributions of (G) the number of localizations/cluster, (H) cluster area, and (I) ICLD show the DEK cluster features differences between control and TSA-treated cells. Panels (J), (K), and (L) show the boxplot representation of the data distributions of the number of localizations per cluster, cluster areas, and ICLDs, respectively, based on SMLM imaging. The average number of localizations per cluster in control cells: 30.7 ± 21.9 compared with TSA-treated cells: 37.5 ± 29.9. The average cluster areas control cells: 2282.8 ± 1131.1 [nm^2^] compared with TSA-treated cells: 2687.3 ± 1450.7 [nm^2^]. The average ICLDs in control cells: 0.0151 ± 0.0106 [N_loc_/cluster] and TSA-treated cells: 0.0156 ± 0.0121 [N_loc_/cluster]. The total number of analyzed clusters is: N_control_ = 22007 and N_TSA-treated_ = 47584. Not significant when p > 0.05; ∗∗ when p ≤ 0.01; ∗∗∗ when p ≤ 0.001. Scale bar: 5 μm. Scale bar insets: 500 nm. See also Figures S2 and S4.

Using SMLM imaging combined with clustering analysis, we evaluated the features of the DEK clusters corresponding to control cells (Figures 3A, 3C and 4A–4C) and TSA-treated cells (Figures 3D–3F and 4D–4F). The clustering analysis provided the distributions of a number of localizations per cluster (Figures 3G and 4G) and the cluster areas (Figures 3H and 4H). We observed a statistically significant increase in the number of localizations per cluster in cells treated with TSA in comparison to control cells (25% and 22.1% for MCF10A and MDAMB231 cells, respectively) together with a statistically significant increase in the mean cluster area (14.3% and 17.7% for MCF10A and MDAMB231 cells, respectively) (Figures S2C–S2F). Moreover, we compared how the population of DEK clusters changes after the TSA treatment considering their areas. We observed a shift in the ratio between “small clusters” and “big clusters” for both cell lines in the case of the TSA-treated samples. The pie charts (Figure S4) provide a visual representation of these changes showing the overall percentage increase of big DEK clusters following the treatment, highlighting the potential alteration in the nanoscale organization.

The analysis of the distributions of the DEK ICLDs (Figure 3I) revealed that there were no statistically significant differences between control and TSA-treated cells for both cell lines. This result suggests that the local density of DEK within clusters is conserved not only regardless of the DEK expression level but also regardless of the chromatin compaction. In other words, despite the nanoscale rearrangement of DEK after TSA treatment demonstrated by the number of localizations per cluster and area differences, the overall ICLD is maintained constant, and this feature is conserved across cell types and conditions, suggesting the preservation of the DEK protein functioning.

## Discussion

It is well established that the DEK protein is overexpressed in highly proliferating cells,^44^ such as cancer cells, and can alter the chromatin topology.^7^ Our recent study using super-resolution techniques suggested that the commonly observed dispersed nuclear DEK pattern^22^^,^^36^ is composed of nanoclusters.^10^ In this work, we investigated the nanoscale characteristics of such clusters in normal-like and aggressive types of breast cancer cell lines to study if DEK’s overexpression correlates with its cluster features. Moreover, we showed how chromatin decompaction influences the DEK clusters in both cell lines.

Over 20 years ago, it has been shown that DEK stays in the ratio of 2 to 3 DEK copies per nucleosome *in vitro*^7^ and 0.25 to 0.5 DEK copy per nucleosome *in vivo*,^4^ depending on the proliferative state of the cell and their cell type. Interestingly, it has also been reported that DEK can multimerize.^45^

Our results show that the number of localizations per cluster depends on the cell type and, more precisely, could be related to the DEK expression level. The literature shows high global overexpression of DEK in MDAMB231 cells with respect to MCF10A.^32^^,^^35^ Our results demonstrate that locally the nano-organization of DEK within clusters is higher by 13% of the average number of localizations in MDAMB231 than in MCF10A cells. Interestingly, even if, in the case of MDAMB231 cells, the DEK clusters exhibit a larger mean area than in MCF10A cells, the difference in their distributions is not statistically significant. However, what we observed was the conservation of DEK protein ICLD in both cell types.

We further showed that the ICLD of DEK is also maintained after the TSA treatment. In the context of breast cancer, TSA has been extensively studied as a molecular target for anticancer therapy.^46^^,^^47^^,^^48^ It has been previously shown that histone modifications are crucial for the regulation of the accessibility of chromatin to DNA-binding factors,^49^ and in cancerous cells, the local chromatin environment can be perturbed.^50^ In the case of global histone acetylation, the opening of the chromatin enhances gene transcription and increases accessibility for nuclear proteins such as RNA polymerase II and other transcription factors.^51^^,^^52^ Studied with super-resolution methods, TSA treatment results in a lower number of localizations per nanodomain and lower nanodomain areas of H2B histone clusters in hyperacetylated human fibroblasts in comparison to non-treated cells.^38^ TSA induces chromatin opening^53^ and a decrease in DNA density associated with nucleosome clutches.^43^ These conditions induce the formation of a large number of newly accessible sites in the genome. Here, in this work, we found that under the global chromatin changes inflicted by the TSA treatment of both cell lines, DEK protein reorganizes into bigger clusters with a higher number of localizations (around 25% and 22% higher number of localizations for MCF10A and MDAMB231 cells, respectively, and around 14% and 18% higher cluster area for MCF10A and MDAMB231 cells, respectively). This finding nicely demonstrates that upon the histone hyperacetylation and increased accessibility to the nucleosomal DNA, chromatin-associated proteins can reorganize into bigger nanostructures. This observation stays in line with other works in which a redistribution of nuclear organization of chromatin-associated proteins has been observed (for example, heterochromatin protein 1 [HP1]).^54^ Furthermore, single-molecule localization imaging demonstrated the association of RNA polymerase II to nucleosome clutches of smaller size and fewer number of nucleosomes, which correspond to the clutches similar to the ones generated after TSA treatment.^38^ It is likely that under the condition of the hyperacetylation of histone tails and DNA decompaction in chromatin, DEK protein accessibility is higher. Indeed, in both investigated cell lines, TSA treatment induced the formation of bigger clusters with a higher number of localizations. Additionally, we found that after the TSA treatment in both cell lines, the ratio between smaller and bigger clusters changed. For MCF10A cells, the total population comprises 31% and 69% of large and small clusters, respectively, in control cells, whereas in cells after the TSA, it is 42% and 58%. We made similar observations for MDAMB231 cells, where the overall population of DEK clusters contains 34% and 66% of large and small clusters in the control sample and 46% and 54% after the TSA incubation. Interestingly, although the area and number of localizations per cluster change upon the hyperacetylation conditions, the intra-cluster localization density of DEK stays conserved.

These semi-quantitative observations suggest that the nano-environment of DEK clusters is maintained and is not influenced by overexpression of the protein itself or by the change of chromatin compaction.

### Limitations of the study

This investigation into the nanoscale arrangement of the DEK protein in breast cancer cell lines and its response to chromatin decompaction induced by Trichostatin A (TSA) treatment can be enhanced in several ways. Firstly, although MCF10A and MDAMB231 cells offer valuable insights, studying a broader range of samples can provide a more comprehensive understanding of the heterogeneity present in breast cancer subtypes. Secondly, despite the power of SMLM technique, it has limitations, urging future quantitative super-resolution studies, possibly incorporating comparative structures like DNA origami for enhanced precision. Additionally, bridging the gap between DEK cluster features and functional implications is a promising research direction. Furthermore, although we examined natural variations in DEK expression, unexplored potential lies in alternative methodologies such as CRISPR-Cas technology for controlled manipulation of DEK expression levels. Lastly, although we acknowledged TSA’s influence on DEK clusters characteristics, we did not fully explore other potential factors (such as cell-cycle phase, specific chromatin states, cell stressors, or external stimuli) that could impact DEK’s nuclear organization.

## STAR★Methods

### Key resources table

**Table:** 

REAGENT or RESOURCE	SOURCE	IDENTIFIER
**Antibodies**		

mouse monoclonal IgG1 κ DEK antibody	Santa Cruz Biotechnology	sc-13622; RRID: AB_2245948
of F(ab')2-Goat anti-Mouse IgG (H+L) Cross-Adsorbed Secondary Antibody, Alexa Fluor 647	Thermo Fisher Scientific	A21237; RRID: AB_2535806

**Chemicals, peptides, and recombinant proteins**		

DMEM : F-12	Gibco™, Thermo Fisher Scientific	11330057
DMEM	Gibco™, Thermo Fisher Scientific	11965092
Penicillin/streptomycin	Sigma-Aldrich	G6784
Insulin	Sigma-Aldrich	I9278
Hydrocortisone	Sigma-Aldrich	H0888
Human Epidermal Growth Factor	Sigma-Aldrich	E9644
Fetal Bovine Serum	Euroclone	ECS0180L
Pork Gelatin	Sigma-Aldrich	G2500
Trichostatin A	Sigma-Aldrich	T8552
ToPro3	Invitrogen™, Thermo Fisher Scientific	T3605
Catalase	Sigma-Aldrich	#C40-100MG
Glucose oxidase	Sigma-Aldrich	G2133-250KU
Cysteamine	Sigma-Aldrich	30070-50G

**Experimental models: Cell lines**		

MCF10A human breast cell line	ATCC	cat# CRL-10317; RRID: CVCL_0598
MDA-MB-231 human breast cell line	ATCC	cat# HTB-26; CVCL_0062

**Software and algorithms**		

Insight3	Dr. Bo Huang of the University of California	N/A
Cluster Analysis code	The MathWorks, Natick, MA	https://www.mathworks.com/products/new_products/release2020b.html
ImageJ/FIJI	http://imagej.nih.gov/ij/	N/A
OriginPro, Version 2020	OriginLab Corporation, Northampton, MA, USA	https://www.originlab.com/2020
GraphPad Prism version 9.4.1	GraphPad Software, San Diego, California USA,	www.graphpad.com

**Other**		

N-STORM TIRF Eclipse Ti2 microscope	Nikon Instruments, Tokyo, Japan	N/A
sCMOS camera	ORCA-Flash 4.0, Hamamatsu Photonics K.K.	N/A

### Resource availability

#### Lead contact

Further information and request for resources and reagents should be directed to and will be fulfilled by the lead contact, Francesca Cella Zanacchi francesca.cella@unipi.it.

#### Materials availability

This study did not generate new unique reagents.

### Experimental model and study participant details

#### Cell lines

MCF10A (cat# CRL-10317) and MDA-MB-231 (cat# HTB-26) cell lines were purchased from American Type Culture Collection (ATCC). MCF10A cells were grown in DMEM : F-12 (Dulbecco's Modified Eagle Medium : Nutrient Mixture F-12) (1 : 1) medium (Gibco, 11330057) supplemented with 5% Horse Serum (HS), 2 mM L-glutamine and 1% penicillin/streptomycin (Sigma-Aldrich, G6784), 10 μg/ml insulin (Sigma-Aldrich, I9278) and 0,5 μg/ml hydrocortisone (Sigma-Aldrich, H0888). Human Epidermal Growth Factor (hEGF, Sigma-Aldrich, E9644) was added freshly before the use of full medium (20 nm/ml). MDA-MB-231 cells were grown in DMEM medium (Gibco, 11965092) supplemented with 10% Fetal Bovine Serum (FBS, Euroclone, ECS0180L), 1% penicillin/streptomycin (Sigma-Aldrich, G6784) and 2 mM L-glutamine. Cells were grown on 10cm^2^ Petri Dish for a month (about 10-12 cell passages) at 37°C in 5% CO_2_.

### Method details

#### Immunolabelling and DNA staining

For any experiments on fixed cells, cells were grown on μ-Slide 8-well with glass bottom (Ibidi GmbH, Germany) coated with 0,5% (w/v) pork gelatin (Sigma-Aldrich, G2500) previously dissolved in phosphate buffer saline (PBS) and autoclaved. For hyperacetylation experiments, MCF10A and MDAMB231 cells were treated with 300 nM of trichostatin A (TSA) (Sigma-Aldrich, T8552) in a complete growth medium for 24h prior to the fixation. For the detection of the protein of interest, the cell cultures were washed with pre-warmed PBS 3x and fixed with formaldehyde (3,7%, methanol free). After the subsequent blocking with the blocking buffer composed of 0,1% Triton X-100 and 3% w/v of bovine serum albumin (BSA) for 1h at room temperature, samples were incubated with the primary antibody (overnight at +4°C) α-DEK (mouse, Santa Cruz, sc-136222, 1:50). Secondary antibodies staining was performed with the use of F(ab')2-Goat anti-Mouse IgG (H+L) Cross-Adsorbed Secondary Antibody, Alexa Fluor 647 (ThermoFisher Scientific, A21237, 1:250, 45min in RT). After finishing the immunostaining, samples were post-fixed in PFA 2% for 5 min and stored in PBS at +4°C. For the experiments in which the DNA in cells was stained, the cells were incubated with the DNA precursor, 5-ethynyl-2'-deoxyuridine (EdU), with the final concentration of 10 μM in complete medium 24h prior to the fixation. For demonstrating the TSA-induced chromatin decondesation ToPro3 dye (T3605, 1:2000 in PBS) was used for 20min incubation prior to the washing and fixation.

#### Single-molecule localization microscopy imaging

Imaging was performed using a commercial N-STORM TIRF Eclipse Ti2 microscope equipped with an oil immersion objective (CFI SR HP Apochromat TIRF 100XC Oil, NA 1.49). Imaging was performed by acquiring 20000 frames with an exposure time of 30 ms using oblique incidence excitation. For the imaging of Alexa Fluor 647, the 647 nm laser was used in order to excite and switch into the dark state of the dye. The 405 nm laser light was used in order to reactivate the dye into a fluorescent state. The imaging was performed with a repeating cycle of 1 activation frame followed by 3 read-out frames, with the use of sCMOS camera (ORCA-Flash 4.0, Hamamatsu Photonics K.K.). All the measurements were performed following the same imaging protocol. The Nikon Perfect Focus System was applied during the entire recording process. The acquisition was performed with the use of a Continuous STORM filter. All samples were imaged in the previously described imaging buffer with the use of the GLOX component.^37^ The buffer contains a glucose oxidase solution as the oxygen scavenging system: 40 mg/ml catalase (Sigma-Aldrich, #C40-100MG), 0.5 mg/ml glucose oxidase (Sigma-Aldrich, G2133-250KU), and MEA 10 mM: cysteamine (Sigma-Aldrich, 30070-50G) in 360 mM Tris-HCl.

#### Image reconstruction and clustering analysis

The images were reconstructed using custom software (Insight3, kindly provided by Dr. Bo Huang of the University of California) by Gaussian fitting of the single-molecule raw images in each frame to determine the x–y coordinates. First, molecules are identified by setting a common threshold of 600 counts/pixel. The Gaussian fitting provides the positions of each identified molecule, and the final images were obtained by plotting each identified molecule as a Gaussian spot. Images were corrected for drift by cross-correlating images obtained from subsets of frames as described in the literature.^55^ Molecules exhibiting a localization precision below 10nm are excluded to avoid aspecific signals and false localizations. Cluster analysis of STORM datasets was performed with a custom-written code (MATLAB, The MathWorks, Natick, MA) implementing a distance-based clustering algorithm previously published.^38^^,^^56^ This density-based clustering algorithm is used to identify spatial localization clusters, with a minimum number of localizations per cluster equal to 20 N_Loc_/cluster to select the DEK contribution and avoid aspecific backgrounds and aggregates. Briefly, a density map is obtained and transformed into binary images. From the binary images, only localizations lying on adjacent (six-connected neighbors) nonzero pixels of the binary image were considered. Initialization values for the number of clusters and the relative centroid coordinates were obtained from the density map identifying local maxima within the connected region, and localizations were associated with clusters based on their distance from cluster centroids. New cluster centroid coordinates were iteratively calculated as the average of localization coordinates belonging to the same cluster. The procedure was iterated until convergence of the sum of the squared distances between localizations and the associated cluster. Selected parameters were kept constant to ensure proper comparison among the different samples.

#### Confocal microscopy imaging

Imaging was performed on a Leica TCS SP5 confocal laser-scanning microscope, using an HCX PL APO 100x/1.40-0.70 oil immersion objective lens (Leica Microsystems, Germany). Excitation was provided with a white light laser (SuperK, NKT) at 642 nm wavelength for ToPro3 dye, with the emission band 650-700 nm. The signal was detected by a Hybrid Detector (HyD). Measurements were performed with 512 x 512 pixel images acquired with a pixel size of 40nm. Confocal images were processed using ImageJ (http://imagej.nih.gov/ij/).

### Quantification and statistical analysis

#### Statistical analysis

The experiments were performed on MCF10A cells (N_MCF10A_ = 10) and MDAMB231 cells (N_MDAMB231_ = 13) in n ≥ 3 independent measurements for each condition, where n refers to a different well in the Ibidi multichamber with fixation, immunolabelling and washing steps performed independently. The total number of analyzed clusters for MCF10A control cells was 26905 (with the average N_clusters/cell_ = 4484 ± 731), for MCF10A TSA-treated cells was 21913 (with the average N_clusters/cell_ = 5479 ± 879), MDAMB231 control cells was 22007 (with the average N_clusters/cell_ = 7336 ± 4396) and MDAMB231 TSA-treated cells was 47584 (with the average N_clusters/cell_ = 7931 ± 3263). Statistical tests were performed in OriginPro, Version 2020 (OriginLab Corporation, Northampton, MA, USA) and GraphPad Prism version 9.4.1 (GraphPad Software, San Diego, California USA, www.graphpad.com). Data points corresponding to the number of localizations, cluster areas, and ICLDs were tested with a non-parametric two-sample, two-tailed Kolmogorov-Smirnov test. In order to obtain the statistical significance level of the differences between the distributions composed of a large set of data (between 22 000 and 47 000 clusters identified), the bootstrapping approach was applied. In brief, 1000 data points were randomly sampled out of each of the datasets (number of localizations, areas, and densities), the maximum difference was obtained by the K-S test, and the process was repeated 1000 times. In that way, a mean difference with the standard deviation was obtained, which has been compared to a p-value. As a control, the data coming from the same distribution (e.g., MCF10A control sample) was sampled as above, resulting in high p-values for intrinsic data. The comparison of the distributions between samples leads to high differences and thus low p-values (not significant, n.s. when p > 0.05; significant when “ ∗ ” means p ≤ 0.05; “ ∗∗ ” means p ≤ 0.01; “ ∗∗∗ ” means p ≤ 0.001). Where the box plots are used on the Figures 1, 3, and 4, the asterisk marks the mean, the line corresponds to the median, box boundaries represent the interquartile range, and the whiskers indicate the minimum and maximum values.

## Data Availability

• The RAW microscopy data within the manuscript will be available from the lead contact upon request • This article does not report the original code • Any additional information in this manuscript is available from the lead contact upon request
