# Supplementary material for: Super-resolution microscopy reveals the nanoscale cluster architecture of the DEK protein cancer biomarker
Source: iScience. 2023 Oct 19;26(11):108277. doi: 10.1016/j.isci.2023.108277 (PMC10660485; doi:10.1016/j.isci.2023.108277)
Supplement: Document S1. Figures S1–S4 [file mmc1.pdf]

**iScience, Volume *26***

## Supplemental information

# Super-resolution microscopy reveals the nanoscale cluster architecture of the DEK protein cancer biomarker

**Agnieszka Pierzynska-Mach, Alberto Diaspro, and Francesca Cella Zanacchi**

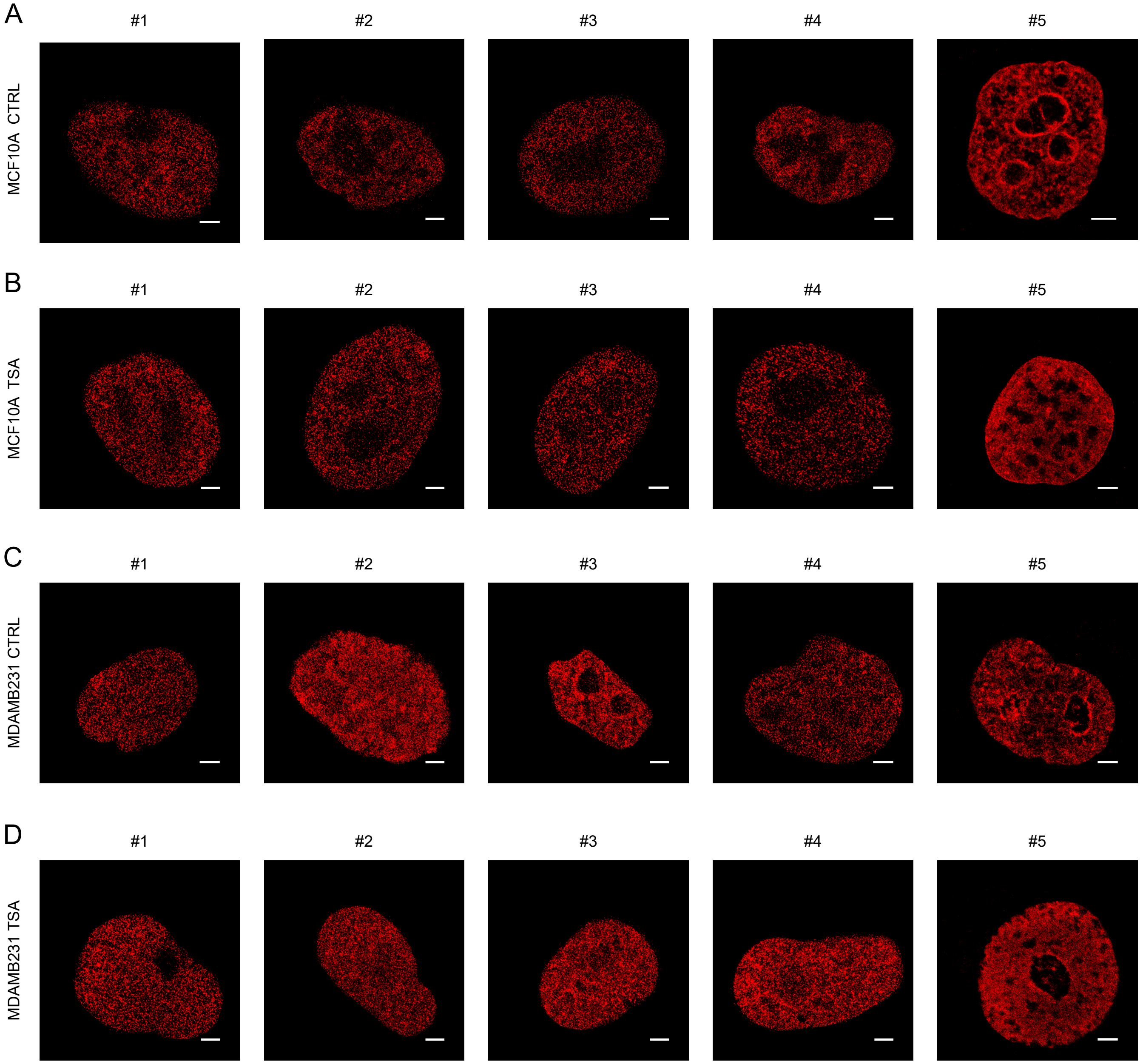


**Figure S1. Nuclear distribution of DEK protein and DNA in each experimental conditions** (related to Figure 1). Reconstructed SMLM images of: (A) MCF10A control cells, (B) MCF10A TSA-treated cells, (C) MDAMB231 control cells, and (D) MDAMB231 TSA-treated cells. For each condition, images from #1 to #4 depict cell nuclei immunolabeled with AF647 against DEK, whereas #5 depicts nuclear DNA marked with EdU and labeled by "click" reaction with AF647 azide. Scale bars: 2000 nm.

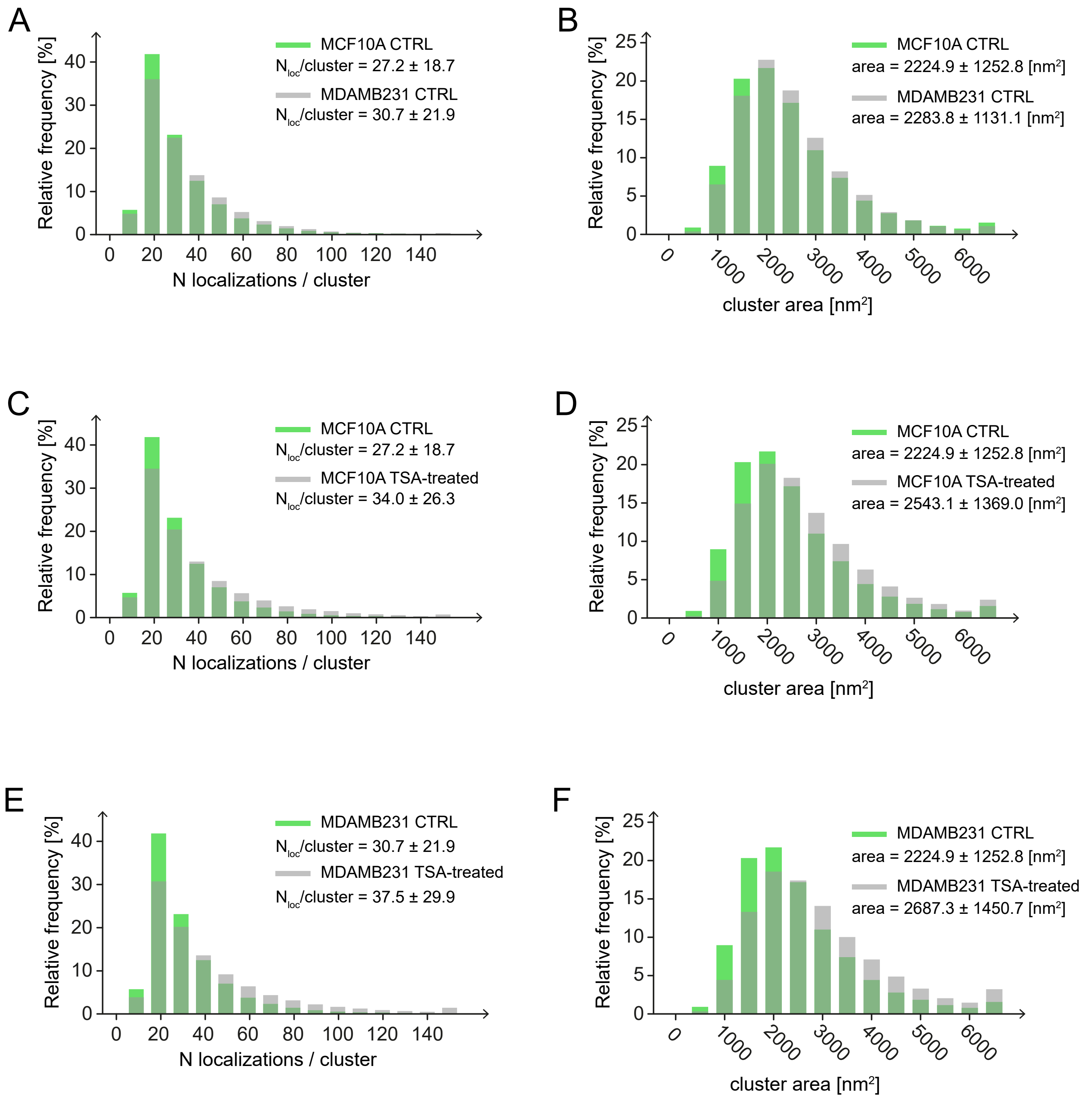


**Figure S2. Frequency distributions of DEK clusters' features based on SMLM imaging** (related to Figure 1, 3 and 4). The cluster parameters were measured in the immunolabelled with AF647 samples and compared between: (A, B) MCF10A and MDAMB231 control cells, (C, D) MCF10A control and TSA-treated cells, and (E, F) MDAMB231 control and TSA-treated cells. The total number of analyzed clusters in MCF10A cells: $N_{control}$ = 26905 and $N_{TSA\text{-}treated}$ = 21913. The total number of analyzed clusters in MDAMB231 cells: $N_{control}$ = 22007 and $N_{TSA\text{-}treated}$ = 47584.

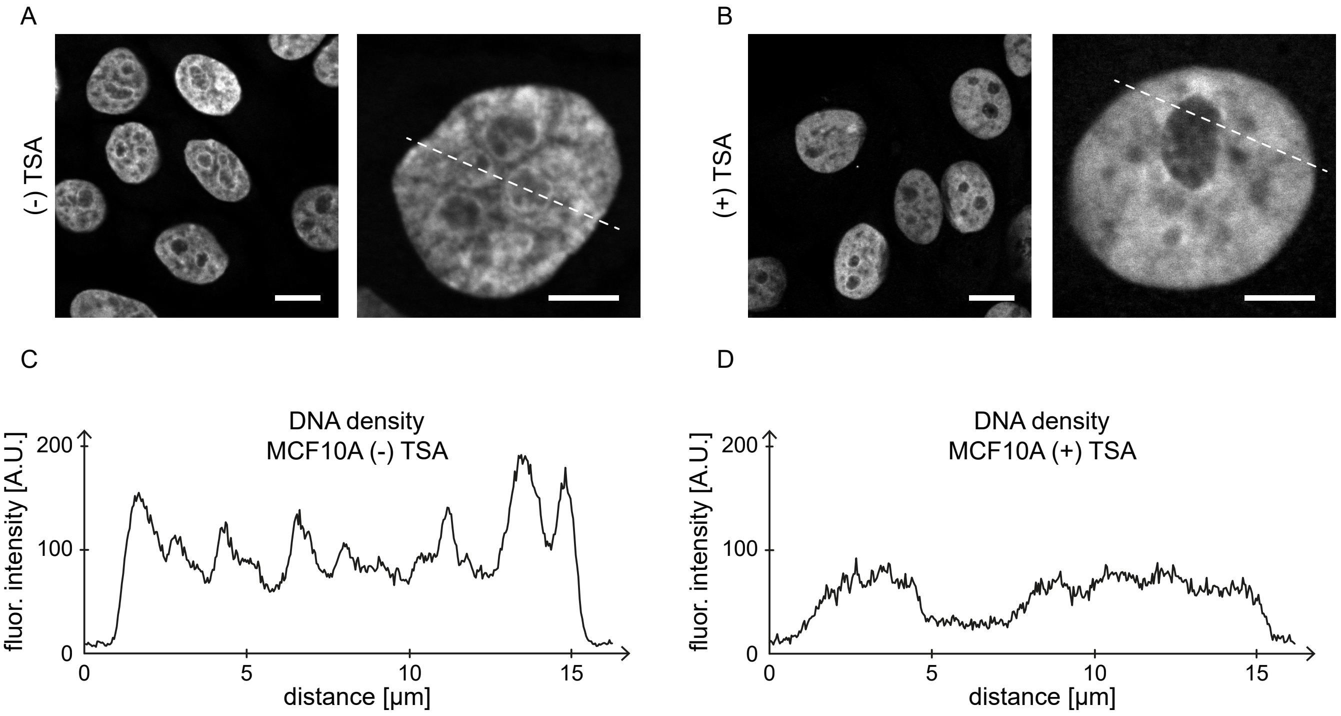


**Figure S3. Chromatin relaxation under the TSA treatment** (related to Figure 2). Confocal images of control (A) and TSA-treated (B) MCF10A cells stained with ToPro3 dye. The variations of measured fluorescence intensity (C, D) reveal the local DNA density. The fluorescence intensity profiles were calculated along the dashed lines. Scale bars in (A) and (B): 10 (left panel) and 5 μm (right panel).

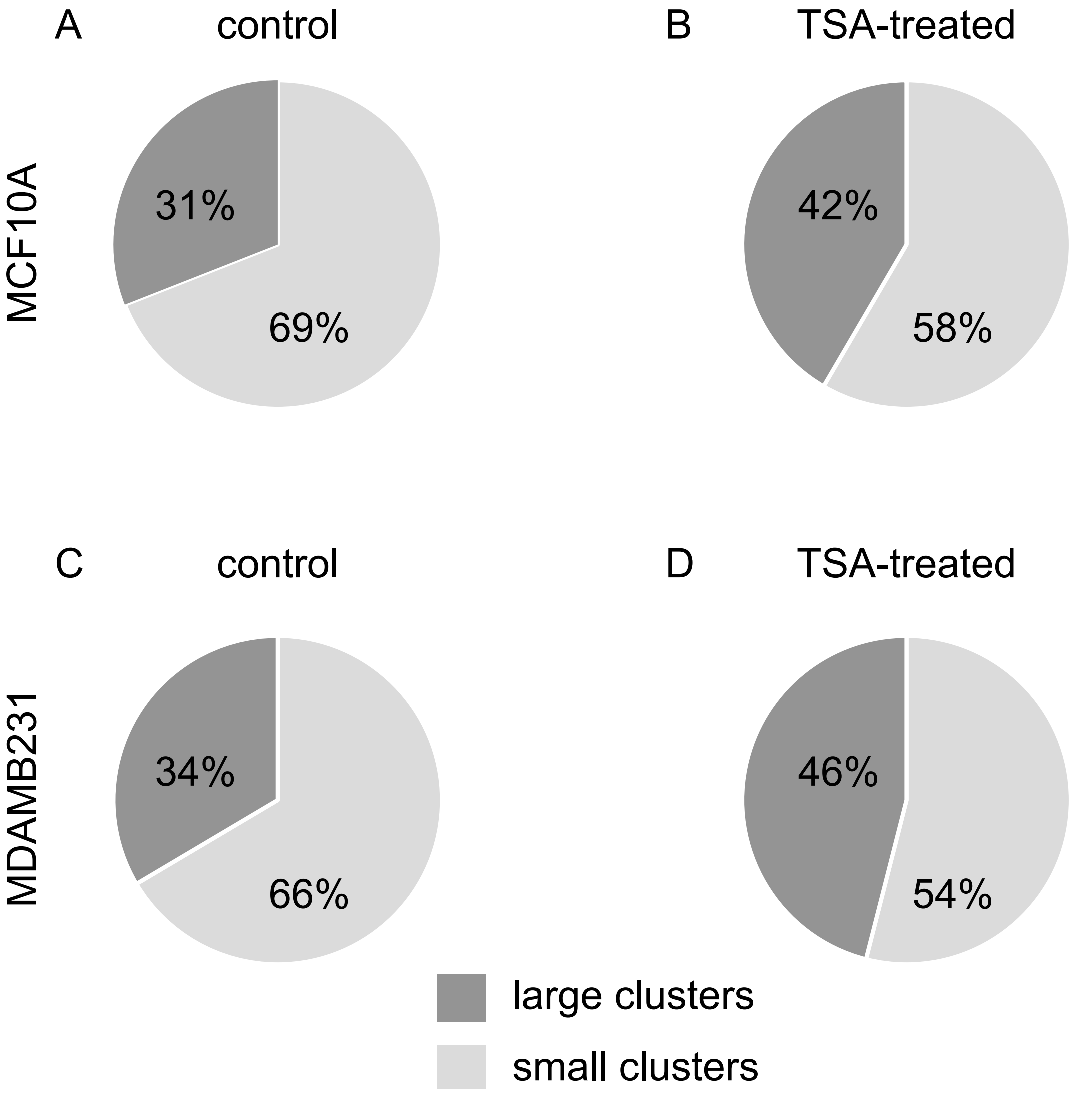


**Figure S4. Comparison of DEK cluster areas ratios between control and TSA-treated cells** (related to Figure 1, 3, and 4). Pie charts illustrate the difference of the area sizes distributions of DEK clusters in control and chromatin-relaxed conditions in MCF10A (A, B) and MDAMB231 (C, D) cells. The analysis is based on a threshold of 2500 square nanometers for classifying clusters as 'small' (below the threshold) and 'large' (above the threshold).
